# Towards the Identification of a Suitable Commercial Diet for Carpione (*Salmo carpio*, Linnaeus 1758): A Multidisciplinary Study on Fish Performances, Animal Welfare and Quality Traits

**DOI:** 10.3390/ani12151918

**Published:** 2022-07-27

**Authors:** Basilio Randazzo, Matteo Zarantoniello, Giulia Secci, Filippo Faccenda, Francesca Fava, Giulia Marzorati, Alessia Belloni, Francesca Maradonna, Veronica Orazi, Roberto Cerri, Michele Povinelli, Giuliana Parisi, Elisabetta Giorgini, Ike Olivotto

**Affiliations:** 1 Department of Life and Environmental Sciences, Polytechnic University of Marche, 60131 Ancona, Italy; b.randazzo.live@gmail.com (B.R.); matteo.zarantoniello@gmail.com (M.Z.); a.belloni@pm.univpm.it (A.B.); f.maradonna@univpm.it (F.M.); v.orazi@yahoo.com (V.O.); e.giorgini@univpm.it (E.G.); 2 Department of Agriculture Food Environment and Forestry, University of Florence, 50144 Firenze, Italy; giulia.secci@unifi.it (G.S.); giuliana.parisi@unifi.it (G.P.); 3 Edmund Mach Foundation, San Michele all’Adige, 38010 Trento, Italy; filippo.faccenda@fmach.it (F.F.); francesca.fava1978@gmail.com (F.F.); marzorati.giulia@gmail.com (G.M.); michele.povinelli@fmach.it (M.P.); 4 Agricola Italiana Alimentare (A.I.A.) S.p.A., 37142 Verona, Italy; roberto.cerri@veronesi.it

**Keywords:** salmonids, aquafeeds, processed animal proteins, gut welfare, histology, fillet quality

## Abstract

**Simple Summary:**

Carpione (*Salmo carpio*, Linnaeus 1758) is an endangered precious endemism of Lake Garda (Northern Italy), the largest Italian lake. To date, several bottlenecks about its culture remain unsolved, including the identification of a proper growth-out diet. In the present study, four different grossly isolipidic, isoproteic, and isoenergetic diets containing ingredients from different origins were used for *S. carpio* culture. Specifically, a diet largely based on marine ingredients, and currently used for carpione farming, was used as control. Three other diets were formulated in order to include relevant percentages of vegetable ingredients or processed animal proteins (at two different inclusion levels). After a three-month feeding trial, fish zootechnical performances, welfare, and flesh quality were evaluated through a multidisciplinary approach, including histology, gene expression, chemical analysis, and Fourier transform infrared spectroscopy (FTIR). This study provided the first insights on carpione physiological responses to different commercial dietary formulations.

**Abstract:**

Carpione (*Salmo carpio*, Linnaeus 1758) is an endangered precious endemism of Lake Garda (Northern Italy), the largest Italian lake. To date, several bottlenecks about its culture remain unsolved, including the identification of a proper growth-out diet. The aim of the present study was to test four different grossly isolipidic, isoproteic, and isoenergetic diets in which the main ingredients had a different origin. Specifically, a diet currently used by local farmers for carpione culture, largely based on marine ingredients, was used as control (CTRL), while the other three diets were formulated by partially replacing marine ingredients with plant ones (VEG) or with different percentages of processed animal proteins (PAP1 and PAP2). The feeding trial was run in triplicate, over a three-month period. No significant differences in growth performance among the experimental groups were observed. However, remarkable histological alterations and inflammatory markers upregulation were observed in VEG group, while PAP inclusion played a role in attenuating inflammation and improving nutrient uptake. Fillet analyses highlighted significant differences in marketable traits and flesh fatty acid composition among the experimental groups, including the reduction of polyunsaturated fatty acids related to PAPs inclusion. In conclusion, PAPs used in the present study promoted *S. carpio* gut health and absorption capacity, while further studies are required to maintain proper quality traits of the final product.

## 1. Introduction

Carpione (*Salmo carpio*, Linnaeus 1758) is a salmonid only found in Lake Garda (Italy) which arouses great concerns due to the rapid wild stock decline observed in the last fifty years [1,2]. Carpione has been described as a Lake Garda endemism [3] and, while phylogenetically related to similar fish species such as Mediterranean brown trout (*Salmo cettii*) and marble trout (*Salmo marmoratus*), it presents peculiar features, including specific feeding habits, mainly relying on planktonic organisms [4]. At present, very few studies on this fish species are available and knowledge on its nutritional requirements in captivity is very limited [5].

Over the last decades, several farming programs attempts have been carried out by local farmers although several challenges, including the identification of a proper diet formulation, hindered an efficient rearing strategy. Currently, intensive *S. carpio* farming relies on the use of aquafeeds intended for other salmonids, and a specific diet for this species does not exist. Diets intended for salmonids are generally composed of a mixture of a wide range of ingredients including fishmeal (FM), plant products (soybean meal, corn gluten meal, canola meal, pea meal) and animal by-products (poultry by-product meal, meat meal, blood meal, hydrolyzed feather meal), which are used depending upon price and availability [6]. FM has been considered the optimal protein source for cultured carnivorous fish diets for decades [7], but its dietary inclusion level has been drastically reduced over the last years due to environmental and economic issues. Presently, in most cases, up to 70% dietary proteins included in fish feeds derive from more sustainable and cheaper sources [8]. Plant-derived ingredients, including soybean, pea, and maize gluten, have been extensively used as major alternative dietary protein sources in aquafeed formulation, due to their high protein content and the large market availability [9,10,11,12,13]. However, the unbalanced amino acids profile, the presence of indigestible carbohydrates and anti-nutritional factors of plant-derived ingredients, together with low levels of essential amino acids and polyunsaturated fatty acids (PUFAs) often lead to detrimental effects in several carnivorous fish species, affecting fish health and growth performances, particularly in salmonids [14,15,16,17]. On the other hand, after the authorization by the EC Regulation No. 56/2013, a range of land-produced feedstuff, named “processed animal proteins” (PAPs) represent a preponderant alternative to vegetable protein sources for aquafeed formulations. PAPs, such as poultry by-product meal, bone meal, blood meal, and hydrolyzed feather meal, present several advantages due to their large availability and low price, high nutrient digestibility, and adequate essential amino acid (EAA) profile, which resembles that of FM [18,19,20]. PAPs have been successfully used in feeds for various fish species, such as tench [21], cobia [22], rainbow trout [23,24], gilthead seabream [25,26], Chinook salmon [27], red drum [28], and hybrid tilapia [29]. The main limitations of PAPs employment in fish feed are represented by the variable composition, depending on the quality of raw materials and processing methods and, in some cases, by the deficiency in methionine and lysine [30]. Moreover, the optimum dietary PAPs inclusion is highly dependent on fish species and the overall diet formulation [30]. As regards dietary lipids, both fish oil and vegetable oils are used in different combinations possibly affecting especially the quality traits of the final product [31]. Therefore, finding the right combination of the ingredients available on the market represents a key issue for feed producers, particularly when new dietary formulations are intended for fish species whose dietary preferences are only partially known.

Farmed animal welfare has also become a crucial aspect in the modern aquaculture, addressing research efforts in finding solutions able to satisfy animal health and to guarantee, at same time, the quality features of the final product [32]. To date, several laboratory techniques are used to assess fish welfare and quality traits. Histology and gene expression analysis represent validated techniques useful in providing information on tissue status and fish physiological responses to experimental dietary formulation, while chemical analysis enables to assess fillet nutritional traits of valuable importance for fish reared for human consumption. Moreover, more recently, spectroscopic techniques are receiving increasing attention as novel approaches in modern aquaculture research and are used to corroborate and improve the general output provided by classic laboratory techniques [33].

In the present study, four different grossly isolipidic, isoproteic, and isoenergetic diets containing proteins from different origins were used for *S. carpio* culture as well as different combinations of fish and vegetable oils. Specifically, a diet largely based on marine protein sources, including FM, and currently used for carpione farming, was used as control. Three other diets were formulated in order to include a predominant protein fraction derived from vegetable ingredients or PAPs (in two different percentage of inclusion). As regards the lipid fraction, the control diet had a higher content of fish oil while the remaining diets were characterized by higher content of vegetable ones.

After a three-month feeding trial, fish zootechnical performances, welfare, and flesh quality were evaluated through a multidisciplinary analytical approach, including traditional and novel techniques such as Fourier Transform Infrared Imaging Spectroscopy (FTIR).

## 2. Materials and Methods

### 2.1. Ethics

All the procedures involving animals were performed according to EU directives on the protection of animal used for scientific purposes. The protocol was approved by the Edmund Mach Foundation (FEM) Ethics Committee (protocol n. 1889/2021).

### 2.2. Experimental Diets

Four commercially available diets intended for salmonids, grossly isoproteic (45.8 ± 1% on dry matter, DM), isolipidic (24.1 ± 1% on DM), and isoenergetic (21.4 ± 0.6 MJ kg^−1^ on DM) were used in the present feeding trial (Table 1). A diet in which the crude protein (CP) fraction was mainly derived from marine ingredients (60% CP) was used as control (CTRL). The other three diets were formulated by partially replacing marine ingredients with plant-derived ones or with processed animal proteins (PAPs) derived from poultry by-products meal (PBM) and swine hemoglobin. More specifically, one diet was formulated by replacing 70% of CP from marine ingredients with vegetable ones (VEG) while the two remaining diets were formulated replacing 56% (PAP1) or 68% (PAP2) of CP from marine ingredients with PAPs. As regards the lipid fraction, the control diet had a higher content of fish oil while the remaining diets were characterized by a higher content of vegetable ones.

Diets were manufactured at AIA Veronesi Holding S.p.A. (Quinto di Valpantena, Verona, Italy) by extrusion in 6 mm pellet size and EAA; minerals and vitamins were added to meet salmonids requirements [34]. Feed stocks were stored in a cold room (+4 °C) until they were used. For DM, CP N × 6.25, and ash contents, feed samples were analyzed according to AOAC International [35], and for the crude lipid content according to Burja et al. [36] (Table 2). The fatty acid (FA) profile of the diet was also assessed by using the method detailed in Section 2.9, and it is reported in Table 2.

### 2.3. Fish Rearing Conditions, Calculation and Sampling

About 1000 fish were obtained from Consorzio Trentino Piscicoltura and transferred to Fondazione Mach (FEM, San Michele all’Adige, Trento, Italy) experimental fish facility. After one month of acclimatation, 480 fish were anaesthetized (100 mg L^−1^ of MS-222; Finquel^®^, Argent Laboratories, Redmond, WA, USA), then they were measured and selected to be uniform in size (initial mean body weight 240.4 ± 47.9 g; initial mean standard-length 26.9 ± 1.5 cm). Fish were randomly subdivided in 12 fiberglass experimental tanks (700 L capacity, 40 fish per tank) and assigned to the four dietary treatments. Tanks were independently supplied with well water, and 30 daily water exchanges were set to replace about 21 m^3^ of water per day. The mean water temperature was 12 ± 0.04 °C (with limited daily variation) and dissolved oxygen was 8.9 ± 0.13 mg L^−1^. During the feeding trial, fish were kept under natural photoperiod day length (May, June, July, mean photoperiod 14 L/10D) at San Michele all’Adige, 46°11′30.404” N,11°8′5.195” E). Fish were hand-fed with the test diets ad libitum, five days a week in three daily meals. The feeding trial lasted 12 weeks.

At the end of the feeding trial, all the fish were anesthetized by tricaine methanesulfonate (100 mg L^−1^ MS-222; Finquel^®^, Argent Laboratories, Redmond, WA, USA) and individual biometry measurements (total length and body weight) were recorded. Three fish per tank (9 fish per dietary treatment) were sacrificed by mean of an overdose of the same anesthetic (MS-222, 400 mg L^−1^) to isolate liver, distal intestine, and fillet samples and stored for the laboratory analysis.

Subsequently, the following zootechnical parameters were calculated, as indicated:

K= [fish weight/(fish standard length)^3^] × 100

SGR = [(ln final body weight-ln initial body weight)/days] × 100

FCR = feed intake per tank/weight gain per tank

PER = weight gain/ingested protein

### 2.4. Intestine and Liver Histology

Liver and distal intestine samples (*n* = 9 for each dietary group) were fixed in Bouin’s solution and stored at 4 °C for 24 h. Samples were than dehydrated by graded ethanol solutions, washed with xylene (Bio-Optica, Milan, Italy), and embedded in solid paraffin (Bio-Optica, Milan, Italy). Sections (5 µm) obtained by a microtome (Leica RM2125RTS, GmbH, Wetzlar, Germany) were stained with Mayer hematoxylin and eosin Y (H&E) (Sigma-Aldrich, Milan, Italy). Alcian blu (Ab) staining was used for mucous cell count in intestine. Slides (ten transversal sections of distal intestine at 200 μm intervals for each sample) were examined under a Zeiss Axio Imager.A2 combined with a color digital camera Axiocam 105 (both from Zeiss, Oberkochen, Germany) microscope, and images obtained were analyzed using ZEN 2.3 (Carl Zeiss Microscopy GmbH) and ImageJ 1.50b (National Institute of Health, Bethesda, MD, USA) software. Distal intestine morphometry was analyzed as previously described by Uran et al. [37]. Mucosal folds height (MF) and submucosa width (SM) were measured on each section. All the undamaged portions were analyzed, and measurements were reported in μm. Mucous cells (MC) abundance was considered as the number of cells counted on 500 of absorptive epithelium, and three random observations were performed on each section. Supranuclear vacuoles (SV), melanomacrophages (MM), and inflammatory influx (Inf) were assessed in two independent blind evaluations as described in Panettieri et al. [38], and an arbitrary unit was assigned based on the incidence of each parameter considered.

The percentage of fat fraction (PFF) in the liver was analyzed, using three histological images for sample per fish (*n* = 27) at 100 μm intervals. Liver histological images were analyzed by means of the ImageJ software 1.50b (National Institute of Health, Bethesda, MD, USA) according to Zarantoniello et al. [39].

### 2.5. Fourier Transform Infrared Imaging (FTIRI) Spectroscopy Measurements and Data Analysis

Liver and muscle biopsies (*n* = 9 for each dietary group) were collected, trimmed at a 5 mm diameter, and immediately stored at −80 °C. As regards fillet, to standardize the sampling, tissue samples were isolated from the same portion, corresponding to the middle region of the fish epaxial muscle.

Three 10 µm-thick sections were obtained using a cryotome, 200 μm apart from each other, and deposited on CaF_2_ optical windows (1 mm thick, 13 mm diameter). Bruker Invenio-R interferometer coupled with a Hyperion 3000 Vis-IR microscope and equipped with a Focal Plane Array (FPA) detector operating at liquid nitrogen temperature (Bruker Optics, Ettlingen, Germany) was used for FTIR analyses. IR maps (164 × 164 µm; 4096 pixel/spectra; 2.56 µm spatial resolution) on each section were acquired in transmission mode in the Mid-InfraRed (MIR) range (4000–800 cm^−1^; spectral resolution 4 cm^−1^; 256 scans). Raw IR maps were pre-processed using the Atmospheric Compensation routine to correct the atmospheric contributions of carbon dioxide and water vapor, and, only for liver samples, the Vector Normalization routine applied on the full frequency range to avoid thickness variations (OPUS 7.5 software package, Bruker Optics, Ettlingen, Germany).

#### 2.5.1. Liver

On each liver section, IR maps were randomly acquired, due to the homogeneity of the samples. False color images represented the topographical distribution of the main biological macromolecules, analyzing the following spectral regions: 3000–2830 cm^−1^ (representative of CH_2_ and CH_3_ groups in lipids chains, LIP); 1760–1724 cm^−1^ (representative of fatty acid regions, FA); 1714–1492 cm^−1^ (representative of amides I and II of proteins, PRT); and 1073–960 cm^−1^ (representative of glycogen, GLY).

For each IR map, the integrated areas of the spectral ranges mentioned above were also calculated and used to obtain the following band area ratios: LIP/PRT, FA/PRT, PRT/TBM, and GLY/PRT; TBM represents the sum of the integrated areas calculated in the 3050–2800 cm^−1^ and 1800–900 cm^−1^ spectral ranges.

#### 2.5.2. Fillet

On each fillet section, IR maps were acquired, and the integrated areas of the following spectral ranges were calculated: 3035–2994 cm^−1^ (representative of = CH groups in unsaturated lipid chains, UNSAT), 2994–2839 cm^−1^ (representative of CH_2_ e CH_3_ groups in lipids chains, LIP); 1770–1713 cm^−1^ (representative of fatty acids, FA); and 1713–1481 cm^−1^ (representative of amides I and II of proteins, PRT). Hence, the following band area ratios were obtained: UNSAT/PRT, LIP/PRT, FA/PRT, and PRT/TBM. TBM was calculated as the sum of the integrated areas of 3050–2800 cm^−1^ and 1800–900 cm^−1^ spectral ranges.

### 2.6. RNA Extraction and cDNA Synthesis

Distal intestine and liver biopsies were prepared according to Olivotto et al. [40,41] and Piccinetti et al. [42]. Total RNA was extracted from the distal intestine and liver samples (*n* = 9 for each experimental group; approximately 90 mg per sample) using the RNAzol^®^ RT reagent (Sigma-Aldrich^®^, R4533, Milan, Italy). A NanoPhotometer^®^ P-Class (Implen, Munich, Germany) and Gel Red™ (Sigma Aldrich, Milan, Italy) staining of 28S and 18S ribosomal RNA bands on 1% agarose gel, were used to assess the RNA concentration and integrity. Complementary DNA (cDNA) was synthesized from 1 µg of total RNA using the LunaScript RT SuperMix Kit (New England Biolabs, Ipswich, MA, USA), and diluted 1:10 in RNase-DNase-free water.. An aliquot of cDNA was used to check the primer pair specificity.

### 2.7. Real Time PCR

The mRNA levels of the genes involved in immune response (intestine samples) such as interleukin-1 (*il6*), interleukin-10 (*il10*), tumor necrosis factor alpha (*tnf-α*), stress (in liver samples), and heat shock protein 70 (*hsp70*) were assessed. Since *S. carpio* transcriptome has not yet been sequenced, a set of primers was designed aligning target sequences of related species (*S. trutta*, *O. mykiss,* and *S. salar*). Primers obtained were used to amplify the target genes analyzed in the present study by using Real Time PCR. Fragments obtained were purified using MinElute^®^ PCR purification Kit (QIAGEN, Hilden, Germany) and sequenced by BMR Genomics (https://www.bmr-genomics.it/ accessed on 31 January 2022). Finally, the fragments’ homology was verified in relation to the sequences of related species already present in NCBI (http://www.ncbi.nlm.nih.gov/ accessed on 31 January 2022). The primers sequences, annealing temperature, and Gene Bank ID are summarized in Table 3. Negative controls revealed no amplification product, and no primer-dimer formation was found in the control templates. PCRs were performed in an iQ5 iCycler thermal cycler (Bio-Rad, Hercules, CA, USA) setting the reaction as follows: 3 min at 95 °C, 45 cycles of 20 s at 95 °C, 20 s at annealing temperature (see Table 4), and 20 s at 72 °C. Fluorescent signal was detected at the end of each cycle, and melting curve analysis was performed to confirm that only one PCR product was present in these reactions.

For the gene expression relative quantification, beta-actin (*β-actin*) and elongation factor 1-α (*ef1-α*) RNA were used as housekeeping genes to standardize the results. Data were analyzed using the iQ5 optical system software version 2.0, including Genex Macro iQ5 Conversion and Genex Macro iQ5 files (all from Bio-Rad). The modification of gene expression was reported with respect to all the groups. Primers were used at a final concentration of 10 pmol μL^−1^.

### 2.8. Physical Characteristics Analyses of Fillets

Fillet and epaxial muscle color and pH values were measured through a Minolta CR-200 Chroma Meter (Konica Minolta, Chiyoda, Japan) and a pH-meter SevenGo SG2™ (Mettler-Toledo, Schwerzenbach, Switzerland), respectively. L* (lightness), a* (redness index), and b* (yellowness index) values were evaluated following the CIELab system [43]. The texture measurement on a portion (3 × 3 cm) of the fillet epaxial muscle was assessed by performing a two-compression test using a ZWICH ROELL^®^ texturometer (Zwick GmnH & Co., Ulm, Germany), fitted with a 10 mm cylindrical stainless-steel probe moving at a constant speed of 30 mm min^−1^ to 50% of the total deformation. Data collection was performed by the testXpert^®^ II V3.0 software (Zwick GmnH & Co., Ulm, Germany) and the results were utilized to calculate the parameters suggested by Veland and Torrissen [44] and Ayala et al. [45]. Afterwards, fillets were skinned, homogenized, and utilized to determine WHC and chemical composition. The water retention capacity of the fillets was determined following the method fully described by Iaconisi et al. [46].

### 2.9. Chemical Characteristics: Proximate Composition, Fatty Acid Profile, and Oxidative Status of the Diets and the Fillets

Moisture, ashes, crude protein, and total lipids of the diets and of the homogenized fillets were determined in accordance with the AOAC method [47]. Total lipids were extracted according to Folch et al. [48], while the FAs were determined in the lipid extract after trans-esterification to methyl esters (FAME), using a base-catalyzed trans-esterification [49] and determined in a Varian GC 430 gas chromatograph (Varian Inc., Palo Alto, CA, USA), furnished with a flame ionization detector and a Supelco Omegawax™ 320 m capillary column (Supelco, Bellefonte, PA, USA). The GC conditions were from the same of Pulido et al. [50]. Chromatograms were recorded using the Galaxie Chromatography Data System 1.9.302.952 (Varian Inc., Palo Alto, CA, USA). FAs were identified by comparing the FAME retention time with those of the Supelco 37 component FAME mix standard (Supelco, Bellefonte, PA, USA) and quantified by means of calibration curves, using tricosanoic acid (C23:0) (Supelco, Belle-fonte, PA, USA) as internal standard.

The results obtained were also utilized to calculate the ratio of the products to the precursor to estimate the Δ5 + Δ6 desaturase activity on both n-3 and n-6 PUFA, following the formula:

Δ5 + Δ6 desaturase (n-3) = [(C20:5-n3 + C22:5n-3 + C22:6n-3)/(C18:3n-3 + C20:5-n3 + C22:5n-3 + C22:6n-3)] × 100

Primary and secondary oxidation products were measured as conjugated dienes (CD) and thiobarbituric acid reactive substances (TBARS), respectively, referring to the spectrophotometric methods [51,52]. Results were expressed as mmol hydroperoxides (mmol Hp) on 100 g fish fillet and malondialdehyde equivalents mg MDA-eq. kg^−1^ fillet, respectively.

### 2.10. Statistical Analysis

Growth performance data are expressed as means and standard error of the means (SEM). Histological measurements, IR, gene expression, and fillet physical and chemical characteristic parameter results were analyzed through a one-way analysis of variance (ANOVA; SAS, 2021) with a Tukey test for the comparison of the means, and data were reported as mean ± standard deviation (SD); the level of significance was set at *p* < 0.05. Data were checked for normal distribution and homogeneity of variance and were analyzed using the SPSS-PC release 17.0 (SPSS Inc., Chicago, IL, USA) and the Graph software package Prism5 (Graph Pad Software, La Jolla, CA, USA).

## 3. Results

### 3.1. Fish Growth Performances

Fish promptly accepted all the tested diets, but the voluntary feed intake did not exceed 1% BW/day during the trial. Morphometric measurements and survival rate did not show statistically significant differences among the experimental groups (*p* > 0.05) after 12 weeks of feeding trial, even though mortality was a little bit higher in fish from the VEG group (Table 4).

Zootechnical indexes (Table 4) did not show statistically significant differences among the groups (*p* > 0.05). Nevertheless, fish from the CTRL group showed the highest SGR and PER and the best FCR compared to the other groups followed by the PAP1 group, which showed the best zootechnical performances if compared to the remaining experimental groups (VEG and PAP2).

### 3.2. Intestine and Liver Histology

**Figure 1:**
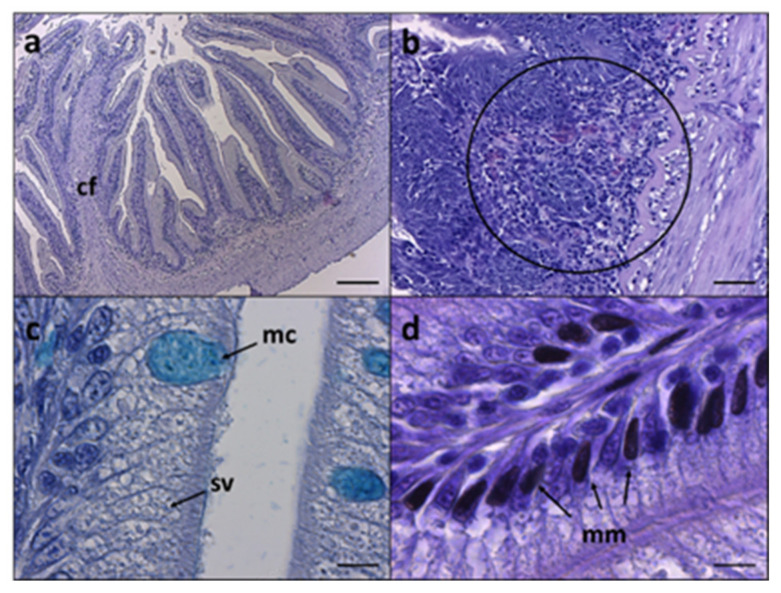
Histological architecture and cell types in *S. carpio* distal intestine. (**a**) Low magnification of intestine mucosa showing a complex fold (cf); (**b**) inflammatory influx in submucosa (circle); (**c**) high-magnification pictures showing mucous cells (mc) and supranuclear vacuoles (sv) and (**d**) melanomacrophages (mm). Stainings: (**a**,**b**,**d**), H&E; (**c**), Ab. Scale bar: (**a**) = 200µm; (**b**) = 50µm; (**c**,**d**) = 10 µm.

**Figure 2:**
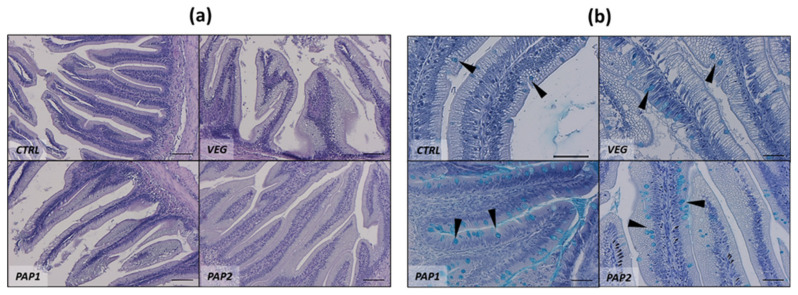
Example of histology of *S. carpio* distal intestine stained with H&E (**a**) and Ab (**b**) from the different experimental groups. Arrow heads indicate mucous cells. Scale bar: (**a**) = 100 µm; (**b**) = 50 µm.

The general histologic architecture of *S. carpio* was similar to that observed in other salmonids species such as rainbow trout and Atlantic salmon (Figure 1). Absorptive mucosa is organized in finger-shaped folds ranging between 400–600 µm length. Among these, branched and up to three times higher folds, already described in other fishes as complex folds [53], were found (Figure 1a); these structures were excluded from the histological morphometric analysis. In the absorptive mucosal layer, MCs were found intercalated among the enterocytes (Figure 1c). Since the enterocytes presented a variable degree of supranuclear cytoplasmatic vacuoles, often resembling MCs shape, Ab staining was useful in discriminating and counting the latter ones (Figure 1c). Moreover, a variable degree of submucosal lymphocyte influx and black, not labelable cells referred to MMs, were observed among the groups (Figure 1d). Examples of distal intestine histologic sections stained with H&E and Ab from all the experimental groups are reported in Figure 2, while mucosal folds morphometry evaluation and histopathological index score analysis are reported in Table 5. Histological morphometry did not show significant differences in MF height among CTRL, PAP1, and PAP2 groups, while a significant MF height reduction was observed in VEG group fish compared to the other groups. Submucosa width was similar among the experimental groups, though slightly thinner in CTRL group fish compared to the other ones. No significant differences in MC number were detected among the experimental groups. Histopathological index score evidenced an increased presence of SV in absorptive mucosa of fish from PAP1 and PAP2 groups. Moreover, while in VEG group fish higher incidence of MM and inflammatory influx was detected, a remarkable reduction of these two indexes was observed in PAP1 group compared to the other groups.

**Figure 3:**
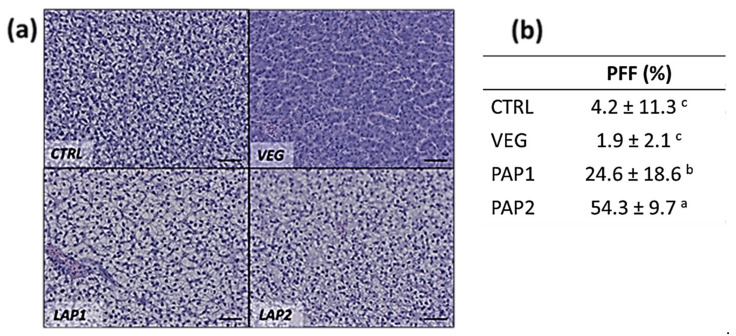
Example of histology of *S. carpio* liver from the different experimental groups (**a**) and the relative percentage of fat fraction (PFF) (**b**). H&E. Scale bar: 50 µm. Different letters indicate statistically significant differences (*p* < 0.05).

All analyzed liver samples showed compact parenchyma, absence of inflammatory events (Figure 3a) and a variable degree of lipid accumulation in relation to diets as shown by the PFF analysed (Figure 3b). Analysis of PFF showed significant differences among the different experimental groups. Particularly PAP1 group presented the highest PFF (54.3 ± 9.7%) compared to the other groups, followed by PAP2 group (24.6 ± 18.6%). Groups fed CTRL and CV diet showed a remarkable reduction of PFF (4.2 ± 11.3% and 1.9 ± 1.9%, respectively) compared to both PAP1 and PAP2 groups. No significant differences in PFF were shown between CTRL and VEG groups, due to the high variability observed in the CTRL group samples.

### 3.3. FTIRI Analysis

#### 3.3.1. Liver

**Figure 4:**
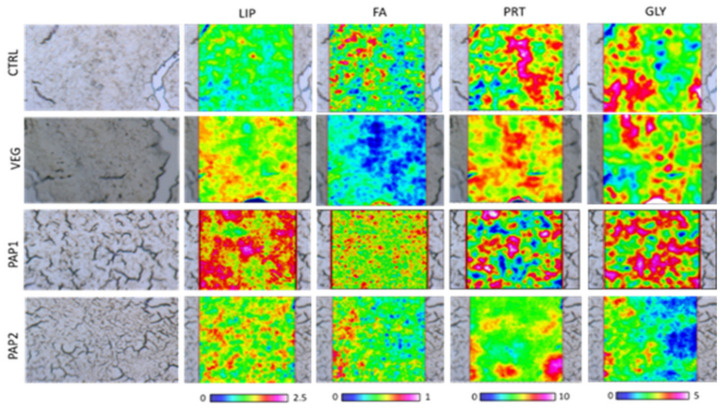
Microphotographs and false color images of representative sections of CTRL, VEG, PAP1, and PAP2 liver samples showing the topographical distribution of lipids (LIP images, scale 0–2.5), of fatty acids (FA images, scale 0–1) proteins (PRT images, scale 0–10), and glycogen (GLY images, scale 0–5) on the mapped areas. IR maps are 164 × 164 μm in size and are composed by 4096 pixel/spectra with a spatial resolution 2.56 × 2.56 μm. A different color scale was adopted: white/light pink indicates high absorbance values of IR radiation, whilst black/dark blue indicates the low ones.

The topographical distributions of lipids, fatty acids, proteins, and glycogen obtained by FTIRI analysis on liver samples are reported in Figure 4, as false color images.

**Figure 5:**
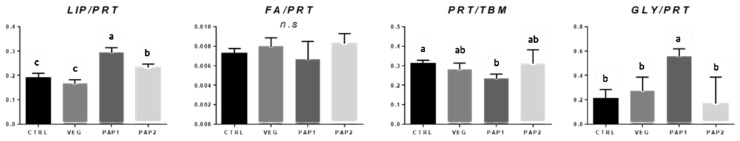
Biochemical composition of liver. Statistical analysis of the following band area ratios: LIP/PRT (relative amount of lipids respect to proteins), FA/PRT (relative amount of fatty acids respect to proteins), PRT/TBM (relative amount of total proteins), and GLY/PRT (relative amount of glycogen respect to proteins). Different letters (a, b, ab, c) indicate statistically significant differences. n.s.: not significant.

To compare the macromolecular composition of liver samples among all experimental diets, specific band area ratios were calculated and statistically analyzed (Figure 5). PAP1 registered statistically significant higher values of both lipids and glycogen, respect to proteins (LIP/PRT and GLY/PRT, *p* < 0.05), and the lowest amount of proteins (PRT/TBM, *p* < 0.05). As regards fatty acids, no statistically significant differences were observed among groups (FA/PRT, *p* > 0.05).

#### 3.3.2. Fillet

**Figure 6:**
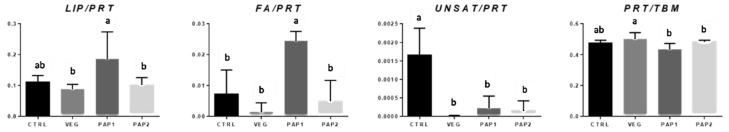
Biochemical composition of fillet. Statistical analysis of the following band area ratios: LIP/PRT (relative amount of total lipids respect to proteins), FA/PRT (relative amount of fatty acids respect to proteins), UNSAT/PRT (relative amount of unsaturated fatty acids respect to proteins), and PRT/TBM (relative amount of total proteins). Different letters indicate statistically significant differences.

The macromolecular composition of muscle samples was investigated by focusing mainly on lipids and proteins (Figure 6). More in detail, the highest amount of lipids and fatty acids were displayed by PAP1 (LIP/PRT and FA/PRT, *p* < 0.05); moreover, among fatty acids, the unsaturated ones were mostly abundant in CTRL (UNSAT/PRT, *p* < 0.05) respect to all the other diets, which showed values close to the zero level (*p* > 0.05). Very tiny differences were observed as regards proteins among groups (PRT/TBM).

### 3.4. Gene Expression

Real-time PCR analyses were performed to test the expression of genes involved in inflammation (il6, il10, and tnf-α) on distal intestine and stress response (hsp70) in liver samples.

**Figure 7:**
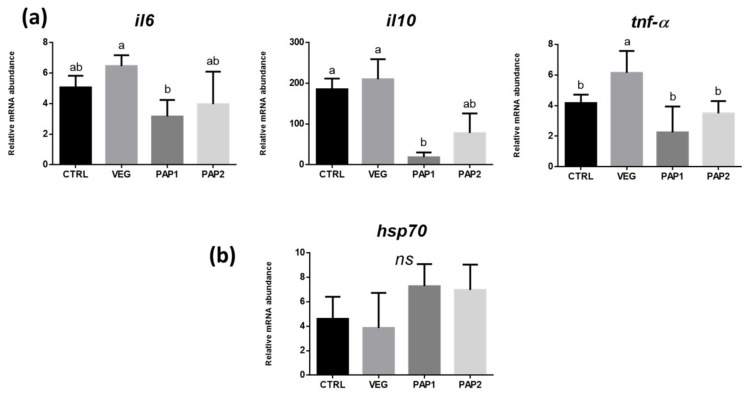
Expression of genes involved in inflammation in distal intestine (**a**) and liver (**b**). Different letters indicate statistically significant differences (*p* < 0.05). Different letters indicate statistically significant differences.

The expression of genes involved in inflammation in the distal intestine was differently modulated by the dietary treatments (Figure 7a). A significant upregulation of all the genes analyzed (*il6*, *il10*, and *tnf-α*) was observed in VEG group fish compared to the others. Notably, a significant *il6* and *il10* gene expression downregulation was observed in PAP1 group compared to the other ones. CTRL and PAP2 groups did not show any significant difference in the expression of *il6* and *il10* compared to the other groups.

No significant differences were observed in *hsp70* gene expression in the liver samples analyzed (Figure 7b). However, though without statistically significant differences, an increase of hsp70 gene expression was observed in liver from PAP1 and PAP2 groups compared to CTRL and VEG groups.

### 3.5. Physical Characteristic Analyses of Fillets

Color analysis (Table 6) was performed on the skin of the fish to assess whether the experimental diets promoted any external changes. It was observed that the experimental diets significantly (*p* < 0.05) affected redness and yellowness indexes, but lightness was not influenced (*p* > 0.05). On one hand, a* was higher in VEG group than in PAP1, while CTRL and PAP2 did not differ from any of the other groups. Concerning the b* index, the presence of PBM at different levels induced a discoloration of fish skin respect to the CTRL and VEG diets. The different diets moderately affected fillet physical characteristics (Table 6). Indeed, fish from VEG and PAP2 groups retained a higher lightness and hardness compared to the CTRL ones (*p* > 0.05), and no other changes emerged.

### 3.6. Fillet Chemical Characteristics: Proximate Composition, Fatty Acid Profile and Oxidative Status of Fillets

Table 7 shows the results of the chemical composition and FA profile of the *S. carpio* fillets. Moisture, ashes, and total lipids of the fillets did not differ (*p* > 0.05) among the dietary groups, whilst the crude protein was significantly (*p* < 0.05) lower in the PAP2 fillets than in the CTRL and PAP1 fillets. Overall, the dietary treatments affected (*p* < 0.05) the proportion of all groups of fatty acids in fillets, except for the saturated fatty acids (SFA). The monounsaturated fatty acids (MUFA) were the most abundant FA class, found highest in the PAP2 fillets. The second most abundant FA group was the n-6PUFA, ranging from 24 to 26 g FA/100 g FAME in the CTRL and PAP2 fillets (*p* < 0.001), respectively. Not surprisingly, the n-3 PUFA content decreased in the diets that were low in marine ingredients, especially in those containing PAPs. The experimental diets affected (*p* < 0.05) the proportion of all fatty acids shown in Table 7, except for palmitoleic acid (C18:1n-7) and dihomo-gamma-linolenic acid (20:3n-6), representing average of 2.90 and 0.79%, respectively. These are two important FAs, as alpha-linolenic (C18:3n-3, ALA), eicosapentaenoic (20:5n-3, EPA), and docosapentaenoic (C22:5n-3) acids were higher in the CTRL and VEG fillets compared to the fillets from fish fed PAPs. Finally, the docosahexaenoic acid (DHA, C22:6n-3) was shortened by decreasing marine ingredients in the diets and its content was the lowest in PAP2 fillets, which also showed the lowest calculated desaturase ability of C18:3n-3.

Concerning the oxidative status, no effect of the dietary treatment was highlighted by the statistical analysis (Table 7).

## 4. Discussion

Currently, *S. carpio* rearing mainly relies on a diet in which substantial portions of marine-derived ingredients are included, while the global trend in aquaculture encourages the use of alternative, more sustainable feeding sources for aquafeed formulations. In the present study, for the first time, the effect of diets in which marine ingredients have been partially substituted with more sustainable ones was tested on carpione zootechnical performances, welfare, and fillet quality traits.

All the tested diets did not impair fish growth at the end of the feeding trial, regardless of the ingredients used to replace the marine-derived ones. This result underlines a good tolerance of this species to all the tested diets in this trial, even when 70% of CP from marine ingredients was replaced with vegetable ones, as observed in VEG group.

This result is in contrast with previous studies performed on salmonids and other species that reported negative effects on fish growth when high amounts of dietary vegetable ingredients are used [15]. However, a simple comparison to other studies could be reductive, since ingredients in fish diets act in synergism and specific feed formulations may change depending on the experimental trial, and a species-specific tolerance to different aquafeed ingredients exists.

The diets tested in the present study are commercially produced, and all of them conserve a percentage of marine-derived ingredients, such as FM and fish oil, which may have had a role in boosting the tolerance of *S. carpio* especially towards vegetable ingredients [54,55,56].

Moreover, the absence of negative side effects on growth in the PAP1 and PAP2 fish groups are in agreement with previous studies, which demonstrated that up to 66% complete replacement of FM with PAPs did not affect growth performances in different carnivorous fish species [57,58,59], including rainbow trout [60], even when PAPs are used in diets completely deprived of FM [24,26].

However, when testing new diets, particular attention must be addressed to intestine integrity since intestinal function is directly linked to feed digestibility/absorption which, in turn, sustains a proper fish growth and welfare [32]. Gut and associated glands, primarily the liver, play a key role in nutrient digestion and absorption as well as in fish immune response [61,62,63].

In the present study, CTRL diet did not lead to significant pathological adverse effects on distal intestine, while a significant reduction of intestine mucosal folds height (turning in a reduced absorptive surface) and an increase in inflammatory influx (including abnormal presence of MM), along with the upregulation of all the inflammatory gene markers analyzed on distal intestine (il6, il10 and tnf-α), were highlighted in fish fed the VEG diet.

Results resemble the widely described symptomatology observed in other salmonids species fed on diets rich in vegetable ingredients [64,65,66] and represent the first evidence that *S. carpio* is also susceptible to the gut inflammatory effects when fed high-vegetable ingredient diets. Within the vegetable-derived ingredients used for VEG diet formulation, soy protein concentrate was exclusively contained in this diet and, together with a general higher content of vegetable-derived ingredients of this diet, this ingredient could have had a role in worsening gut inflammatory condition compared to the other diets [67].

On the contrary, PAPs diets resulted in a better gut condition compared to the others, consisting of a reduction of inflammatory influx and a positive modulation of inflammatory gene expression. Remarkably, a more significant downregulation of inflammatory markers (il6 and il10) was observed in the PAP1 group compared to the PAP2 group. Since in PAP1 group a lower percentage of FM was replaced with PAPs, the right ratio between the two protein sources may have turned in improved gut inflammatory status compared to all the other groups, at least on a molecular level.

Moreover, the increase in enterocytes supranuclear vacuolization in the distal intestine from the PAPs groups indicates a further improvement in gut functionality in fish from these groups [68,69]. Enterocytes supranuclear vacuolization, when not extremely exacerbated, represents a normal condition indicating nutrient pinocytotic uptake; however, a strong reduction is usually associated to an inflamed gut, particularly in salmonids [70].

On the other hand, the diets tested in the present study affected liver composition differently. Particularly, while CTRL and VEG diets lead to a low percentage of lipid deposition in liver parenchyma, both PAPs inclusions caused a higher, dose-dependent lipid accumulation in liver, along with a higher amount of total fat in the fillet (as detected by both chemical and spectroscopical, FTIR, analysis). According to this, Panicz and colleagues [71] observed that tench fed diets in which FM was partially (25.7, 48.6, and 71.4%) or totally replaced by PBM showed increased fat deposition in the muscle tissue.

Such alteration of lipid metabolism could cause a stress response, which can be here observed by the tending higher (although not statistically significant due to the elevated variability among the specimens) hsp70 gene expression in the PAP1 and PAP2 groups’ livers respect to CTRL and VEG ones, as previously demonstrated in different fish species [72].

Since the diets utilized in the present study were formulated to be grossly isolipidic (ranging from 22.9% of CTRL diet to 24.4% of PAP2 diet) using different proportions of fish or vegetable oils, such differences in liver and fillet lipid composition can be attributed to the FA composition of the dietary lipid fraction. Specifically, PAP2 diets showed a higher amount of MUFAs and a higher n-6/n-3 ratio compared to the CTRL and VEG ones, which has been previously related to steatosis onset in freshwater fish species [73,74], and to a high percentage of fat in the fillet [75].

Moreover, while the VEG diet marginally affected fillet FAs profile compared to CTRL one, a decrease in EPA (C20:5n-3) and DHA (C22:6n-3) content was observed in PAP1 and particularly in PAP2 fillets compared to the CTRL ones, consistently with the lower amount of these FAs in the corresponding diets. Fresh water and certain euryhaline fish species, such as salmonids, are able to synthesize PUFAs starting form shorter-chain precursors through the elongation and desaturation pathways [76]. While considering the estimated activity of Δ5 and Δ6 desaturases on n-3 and n-6 PUFAs (Table 7), the values obtained in the present trial were lower than those proposed for rainbow trout by Bruni et al. [77], underlying a reduced ability of carpione in converting shorter FAs precursors in longer n-3 PUFAs.

Wild *S. carpio* mainly feed on planktonic organisms [4] naturally rich in long-chain PUFAs. At the present, no information on desaturation and elongation enzymatic capacity of this fish species are available. Thus, further studies are necessary to better elucidate this aspect.

The present trial revealed a marginal effect of the tested diets on the other quality parameters analyzed, with skin color and fillet hardness being the only significantly modified parameters. Skin and flesh external characteristics are of paramount importance for consumers, and feeding strategies aimed in managing fish color are reported for several finfish such as sparids and salmonids [78,79] due to the strategic role of dietary pigments (such as carotenoids) in tissue coloring. As reviewed by de Carvalho and Caramujo [80], several fish species are able to accumulate carotenoids in the integument of fish skin even if the predominant pigment is peculiar of each fish species. In addition, salmonids are also able to store astaxanthin in muscle giving to the fillet the pink-red aspect highly appreciated by consumers. As previously demonstrated [79], the effect of the diet was more pronounced for skin color than for fillets, with both a* and b* values being significantly increased by the VEG diet probably because of the species-specific interaction between the carotenoids content of the diets and fish metabolism, which needed to be further explored.

Finally, as regards the textural items, the hardness of the fillets was increased in VEG and PAP2 fillets. Similarly, higher shear forces were observed in fillets of fish fed plant-based diets [81,82] or supplemented with high levels of PBM [71].

## 5. Conclusion*s*

Vegetable-derived ingredients in the diet affected *S. carpio* gut welfare comparably to other salmonid fish species, while FM and FO may act as adjuvant in counteracting negative side effects of these ingredients. On the contrary, PAPs used in the present study were able to promote *S. carpio* welfare by promoting gut health and absorption capacity, but their use must be further optimized to guarantee an adequate fillet FAs profile, in order to improve the final quality traits and the consumers’ acceptance towards a valuable food product fed on aquafeeds containing more sustainable ingredients.

## Figures and Tables

**Table 1:** Ingredients used for the formulation of the commercial diets tested in this study. The percentages of inclusion are not included in the table due to business secrecy; however, the ingredients are sorted in descending order based on their amount.

Ingredients		CTRL	VEG	PAP1	PAP2
		Fish meal	Fish meal	PBM proteins	PBM proteins
		Dehulled soy bean meal	Dehulled soy bean meal	Fish meal	Fish meal
		Corn gluten	Wheat gluten	Soy oil	Feather hydrolyzed meal
		Fish oil	Soy oil	Swine hemoglobin	Wheat meal
		Sunflower seeds meal	Fish oil	Wheat meal	Sunflower seeds meal
		Wheat meal	Wheat meal	Fish oil	Fish oil
		Soy oil	Guar germ flour		Rapeseed oil
			Sunflower seeds meal		Swine hemoglobin
			Soy protein concentrate		Soy oil
*Vitamin* *(kg^−1^)*	Vit. A (U.I.)	12,000	11,000	15,000	11,000
	Vit. D3 (U.I.)	2000	1800	2500	1800
	Vit. E (mg)	160	150	200	150
	Vit. C (mg)	160	150	200	150
	Mn (mg)	45	45	50	38
*Minerals* *(kg^−1^)*	Zn (mg)	60	60	75	55
	Fe_2_ (mg)	20	20	26	20
	Cu_2_ (mg)	9	8	12	8
	I (mg)	2	2	3	2
	Se (µg)	160	150	200	150

**Table 2:** Proximate composition (% as DM), gross energy (MJ kg^−1^ as DM) and fatty acid profile (g 100 g^−1^ total FAME) of the experimental diets.

Proximate Composition	CTRL	VEG	PAP1	PAP2
*Dry matter (%)*	91.9	92.5	94.2	94.3
*Crude protein (%)*	46.7	45.4	44.6	46.7
*Crude lipid (%)*	22.9	23.8	25.5	24.4
*Ash (%)*	8.6	6.7	9.6	8.6
*Carbohydrates ^1^ (%)*	21.9	24.1	20.3	20.4
*Gross energy (MJ kg^−1^)*	21.2	20.6	22.1	21.7
**Fatty acids**				
C14:0	2.33	2.06	1.76	1.32
C16:0	13.93	14.00	14.72	12.60
C16:1n-7	2.93	2.39	2.36	2.12
C18:0	3.92	4.20	4.65	4.00
C18:1n-9	28.52	24.02	27.57	38.96
C18:1n-7	2.48	2.12	2.13	2.62
C18:2n-6	25.1	32.12	31.49	24.77
C18:3n-3	4.18	4.45	4.48	4.67
C18:3n-6	0.66	0.60	0.49	0.24
C20:1n-9	1.44	1.17	1.01	1.34
C20:5n-3	3.194	3.036	1.999	1.067
C22:6n-3	5.017	3.906	2.543	1.696
∑ SFA	21.524	21.612	22.458	19.225
∑ MUFA	36.920	31.279	34.353	46.313
∑ *n*-6PUFA	26.312	33.155	32.299	25.669
∑ *n*-3PUFA	14.268	13.010	10.197	8.325

^1^ Calculated by differences as 100—(Moisture + Crude protein + Crude lipid + Ash); SFA: saturated fatty acids; MUFA: monounsaturated fatty acids; PUFA: polyunsaturated fatty acids. The following FAs, found below 0.5% of the total FAME, were utilized for calculating the Σ classes of FAs but are not listed in the table: C12:0, C13:0, C14:1n-5, isoC15:0, C15:0, isoC16:0, C16:1n-9, C16:2n-4, C16:3n-4, C16:4n-1, C17:0, C17:1, C18:2n-4, C18:3n-4, C18:4n-1, C20:0, C20:1n-11, C20:1n-7, C20:3n-3, C20:4n-3, C21:5n-3, C22:0, C22:1n-7, C22:1n-9, C22:2n-6, C22:4n-6, C22:5n-6, C24:0.

**Table 3:** List of primers, sequences, annealing temperature (A.T.), and GeneBank ID used in the present study for gene expression analysis on distal intestine and liver. hk = housekeeping genes.

Gene	Primers Sequences		A.T. °C	Gene Bank ID
	Forward	Reverse		
*il6*	ACTCCCCTCTGTCACACACC	GGCAGACAGGTCCTCCACTA	58	DQ866150
*il10*	CCCAGAGGCCGTACATTTGA	ATTTGTGGAGGGCTTTCCTT	57	AB118099
*tnf-α*	GCTATTCGGACTCCATCGGG	CCCTCGCCGATATTGGACTC	59	NM001124374
*hsp70*	ACCACACCCAGTTATGTCGCCT	CTTCCGCCCTATCAGCCGC	60	AY423555
*β-actin* (hk)	ATGGAAGATGAAATCGCCGCAC	TGGCCCATCCCAACCATCAC	60	AJ438158
*ef1-α* (hk)	GAATCGGCTATGCCTGGTGAC	GGATGATGACCTGAGCGGTG	60	BG933853

**Table 4:** Zootechnical performances of *S. carpio* fed the diets tested over 12 weeks.

Zootechnical Performences	CTRL	VEG	PAP1	PAP2	Significance
*Lenght (cm)*	29.19 ± 1.55	29.19 ± 1.76	29.18 ± 1.85	29.20 ± 1.68	n.s.
*Final weight (g)*	343.3 ± 63.4	340.9 ± 67.3	336.2 ± 75.7	335.8 ± 65.9	n.s.
*K index*	1.37 ± 0.14	1.35 ± 0.10	1.33 ± 0.13	1.34 ± 0.13	n.s.
*Survival (%)*	100	93.3 ± 3.82	99.2 ± 1.44	97.5 ± 2.5	n.s.
*SGR*	0.44 ± 0.04	0.39 ± 0.06	0.40 ± 0.04	0.38 ± 0.03	n.s.
*FCR*	0.98 ± 0.09	1.08 ± 0.07	1.03 ± 0.08	1.11 ± 0.08	n.s.
*PER*	2.39 ± 0.21	2.22 ± 0.27	2.33 ± 0.19	2.06 ± 0.15	n.s.

SGR: specific growth rate; FCR: feed conversion ratio; PER: protein efficiency ratio. n.s.:not statistically significant differences.

**Table 5:** Morphometric evaluation and histological index scores in distal intestine

Histological Morphometry and Index Score	CTRL	VEG	PAP1	PAP2
MF (µm)	562.6 ± 131.4 ^ab^	460.8 ± 11.6 ^b^	592.0 ± 66.8 ^ab^	597.5 ± 142.1 ^ab^
SM (µm)	30.6 ± 11.6 ^ab^	34.5 ± 14.8 ^a^	34.8 ± 13.6 ^a^	25.8 ± 10.4 ^b^
MC (n 500 µm^−1^)	7.8 ± 3.5	9.2 ± 4.1	11.3 ± 6.0	7.9 ± 3.7
SV (a.u.)	+	+	++	++
MM (a.u.)	++	+++	+	+
Inf (a.u.)	++	+++	+	+

MF: mucosal fold height; SM: submucosa width; MC: mucous cells number; SV: supranuclear vacuoles; MM: melanomacrophages; Inf: inflammatory influx. Folds height and submucosa width are expressed by the means of the measurements performed ± SD. Mucous cell count is present as the mean of cells counted (± SD) on 500 µm of absorptive epithelium. SV, MM, and Inf score is reported by mean of an arbitrary unit, assigned based on the incidence of each parameter considered. Different superscript letters indicate significant differences among the experimental groups (a,b: *p* < 0.05).

**Table 6:** Physical characteristics of the skin and fillet of *S. carpio* fed the different dietary treatments. Different letters indicate statistically significant differences (*p* < 0.05).

Fish Portion	Characteristics	CTRL	VEG	PAP1	PAP2	Significance
Fish skin	L*	58.30 ± 5.61	56.23 ± 6.39	56.20 ± 6.76	55.73 ± 8.02	n.s.
	a*	−1.37 ^ab^ ± 0.77	−0.79 ^a^ ± 1.05	−2.08 ^b^ ± 1.03	−1.09 ^ab^ ± 1.25	0.010
	b*	8.86 ^a^ ± 2.65	8.82 ^a^ ± 1.93	6.42 ^b^ ± 1.73	6.54 ^b^ ± 2.06	0.003
Fish fillet	L*	50.79 ^b^ ± 2.15	53.35 ^a^ ± 2.60	51.39 ^ab^ ± 2.07	53.50 ^a^ ± 3.41	0.011
	a*	1.21 ± 1.51	2.28 ± 1.78	2.00 ± 2.39	2.68 ± 1.57	n.s.
	b*	3.23 ± 0.99	3.16 ± 1.65	2.48 ± 1.32	3.19 ± 1.74	n.s.
	WHC, %	91.18 ± 3.81	92.64 ± 3.80	92.28 ± 5.07	90.54 ± 3.79	n.s.
	Hardness, N	3.40 ^b^ ± 1.25	5.09 ^a^ ± 2.24	3.25 ^b^ ± 1.18	5.27 ^a^ ± 1.71	0.001
	Adhesiveness, N mm^−1^	0.67 ± 1.06	0.42 ± 0.31	0.24 ± 0.16	0.41 ± 0.32	n.s.
	Cohesiveness	0.28 ± 0.14	0.22 ± 0.05	0.24 ± 0.05	0.21 ± 0.04	n.s.
	Resilience	0.04 ± 0.05	0.04 ± 0.05	0.04 ± 0.03	0.02 ± 0.02	n.s.

Color parameters are indicated by superscript asterisks (^*^)^.^ WHC: water holding capacity. n.s.: not significant (*p* > 0.05); a,b: means with different superscript letters are significantly different (*p* > 0.05); RMSE: Root Mean Square Error.

**Table 7:** Moisture, ashes, crude protein, total lipids (g 100 g^−1^ of fillet), fatty acid profile (g FA 100 g^−1^ FAME), conjugated dienes (CD, mmol Hp 100 g^−1^ fillet), and TBARS content (mg MDA-eq. kg^−1^ fillet) of fillets from *S. carpio* fed the different dietary treatments.

Fillet Composition	CTRL	VEG	PAP1	PAP2	Significance
Moisture	68.68 ± 1.12	68.63 ± 3.30	66.86 ± 2.61	67.41 ± 4.07	n.s.
Ashes	1.39 ± 0.08	1.34 ± 0.17	1.59 ± 0.62	1.30 ± 0.10	n.s.
Crude protein	20.09 ^a^ ± 0.95	18.99 ^ab^ ± 1.16	19.96 ^a^ ± 1.74	18.07 ^b^ ± 1.16	0.0001
Total lipids	8.47 ± 1.01	9.43 ± 2.82	9.16 ± 3.05	11.24 ± 4.42	n.s.
Fatty acids					
C14:0	1.93 ^a^ ± 0.10	1.88 ^ab^ ± 0.13	1.78 ^bc^ ± 0.07	1.74 ^c^ ± 0.12	<0.0001
C16:0	12.21 ^ab^ ± 0.66	11.97 ^ab^ ± 0.72	12.49 ^a^ ± 0.41	11.87 ^b^ ± 0.67	0.042
C16:1n-7	2.87 ± 0.17	2.88 ± 0.34	2.87 ± 0.33	2.97 ± 0.42	n.s.
C18:0	3.13 ^b^ ± 0.13	3.23 ^ab^ ± 0.17	3.33 ^a^ ± 0.10	3.19 ^b^ ± 0.12	0.001
C18:1n-7	2.72 ^a^ ± 0.04	2.62 ^b^ ± 0.07	2.61 ^b^ ± 0.08	2.76 ^a^ ± 0.06	<0.0001
C18:1n-9	31.81 ^b^ ± 0.78	31.62 ^b^ ± 1.10	31.90 ^b^ ± 1.10	35.50 ^a^ ± 0.61	<0.0001
C18:2n-6	21.60 ^b^ ± 0.80	23.26 ^a^ ± 0.74	23.52 ^a^ ± 0.99	20.98 ^b^ ± 0.86	<0.0001
C18:3n-3	3.45 ^a^ ± 0.15	3.44 ^a^ ± 0.11	3.35 ^ab^ ± 0.11	3.29 ^b^ ± 0.17	0.007
C18:3n-6	0.49 ^b^ ± 0.08	0.53 ^b^ ± 0.06	0.70 ^a^ ± 0.08	0.69 ^a^ ± 0.14	<0.0001
C18:4n-3	0.77 ^ab^ ± 0.06	0.73 ^b^ ± 0.05	0.82 ^a^ ± 0.06	0.79 ^a^ ± 0.07	0.002
C20:1n-9	1.81 ^ab^ ± 0.06	1.80 ^b^ ± 0.11	1.64 ^c^ ± 0.12	1.90 ^a^ ± 0.09	<0.0001
C20:2n-6	1.02 ^ab^ ± 0.06	1.05 ^a^ ± 0.08	0.98 ^bc^ ± 0.08	0.93 ^c^ ± 0.06	0.0003
C20:3n-6	0.75 ± 0.10	0.77 ± 0.05	0.81 ± 0.06	0.82 ± 0.10	n.s.
C20:4n-6	0.47 ^b^ ± 0.04	0.52 ^a^ ± 0.04	0.50 ^ab^ ± 0.05	0.51 ^ab^ ± 0.06	0.038
C20:5n-3	1.58 ^a^ ± 0.08	1.54 ^a^ ± 0.16	1.34 ^b^ ± 0.09	1.26 ^b^ ± 0.21	<0.0001
C22:1n-11	0.66 ^ab^ ± 0.06	0.70 ^a^ ± 0.10	0.59 ^c^ ± 0.06	0.62 ^bc^ ± 0.06	0.0003
C22:5n-3	0.65 ^a^ ± 0.06	0.62 ^a^ ± 0.07	0.56 ^b^ ± 0.03	0.53 ^b^ ± 0.06	<0.0001
C22:6n-3	8.59 ^a^ ± 0.71	7.53 ^b^ ± 0.91	7.08 ^bc^ ± 0.53	6.43 ^c^ ± 0.86	<0.0001
Σ SFA	18.13 ± 0.87	17.92 ± 0.99	18.43 ± 0.51	17.64 ± 0.88	n.s.
Σ MUFA	40.75 ^b^ ± 0.76	40.48 ^b^ ± 1.31	40.44 ^b^ ± 1.60	44.64 ^a^ ± 1.12	<0.0001
Σ n-6PUFA	24.58 ^b^ ± 0.63	26.41 ^a^ ± 0.83	26.79 ^a^ ± 1.09	24.21 ^b^ ± 0.81	<0.0001
Σ n-3PUFA	15.78 ^a^ ± 0.76	14.44 ^b^ ± 1.19	13.72 ^bc^ ± 0.72	12.85 ^c^ ± 1.19	<0.0001
Δ5+Δ6 desaturase (n-6)	6.45 ^a^ ± 0.49	6.32 ^a^ ± 0.37	5.92 ^b^ ± 0.30	6.44 ^a^ ± 0.33	0.0007
Δ5+Δ6 desaturase (n-3)	75.72 ^a^ ± 1.68	73.67 ^b^ ± 2.02	72.80 ^bc^ ± 1.28	71.20 ^c^ ± 2.24	<0.0001
CD	0.187 ± 0.017	0.195 ± 0.053	0.203 ± 0.043	0.226 ± 0.071	n.s.
MDA	0.460 ± 0.379	0.378 ± 0.243	0.411 ± 0.153	0.434 ± 0.245	n.s.

SFA: saturated fatty acids; MUFA: monounsaturated fatty acids; PUFA: polyunsaturated fatty acids; RMSE: Root Mean Square Error. n.s.: not significant (*p* > 0.05); a,b, bc, c: means with different superscript letters are significantly different (*p* < 0.05) among dietary groups. The following FAs, found below 0.5% of the total FAME, were utilized for calculating the Σ classes of FAs but are not listed in the table: C12:0, C13:0, C14:1n-5, isoC15:0, C15:0, isoC16:0, C16:1n-9, C16:2n-4, C16:3n-4, C16:4n-1, C17:0, C17:1, C18:2n-4, C18:3n-4, C18:4n-1, C20:0, C20:1n-11, C20:1n-7, C20:3n-3, C20:4n-3, C21:5n-3, C22:0, C22:1n-7, C22:1n-9, C22:2n-6, C22:4n-6, C22:5n-6, C24:0.

## Data Availability

The data presented in this study are available on request from the corresponding author.
